# Enhancing corporate readiness for and resilience to future public health threats, development and deployment of the public health readiness and resilience (PHRR) assessment tool

**DOI:** 10.3389/frhs.2023.1187229

**Published:** 2023-07-25

**Authors:** Susan Garfield, Rebeca Almeida, Kira Donaldson, Natasha Eslami

**Affiliations:** ^1^ Global Markets/Client Service, Ernst & Young LLP, Cambridge, MA, United States; ^2^ Transformation Architecture, Ernst & Young LLP, New York, NY, United States; ^3^ Transformation Architecture, Ernst & Young LLP, Los Angeles, CA, United States; ^4^ Transformation Architecture, Ernst & Young LLP, Cambridge, MA, United States

**Keywords:** business assessment, public health resilience, public health readiness, corporate assessment, public health framework, corporate resilience, resilience, corporate

## Abstract

As the world suffered through the COVID-19 pandemic, it is increasingly clear that the health of populations is foundational to a high-functioning economy, corporate well-being, and a core driver of social justice. Thus, companies need to understand how to become more resilient to current and future threats. This study (1) explored dimensions of resilience from a public health risk-specific lens and reviewed existing evaluation tools and frameworks to develop a methodology and framework (Public Health Readiness and Resilience—PHRR Assessment Tool) for organizations; and (2) leveraged the framework to evaluate a sample of large corporations to validate the insights the tool can provide, confirm functionality, and evaluate the ability to leverage publicly available vs. propriety data to complete the assessment. We conducted a non-exhaustive search for relevant indices using key word searches and cascade sampling. For the initial review of indices (*n* = 24), the team evaluated each document based on predefined criteria. Gaps identified in the available indices informed the development of the PHRR assessment tool. The tool was then used to examine real-world companies (*n* = 22) from eight different industries. Findings from the PHRR tool illustrated variation in readiness and resilience as well as the availability of data. Approximately half of the companies analyzed (*n* = 11) indicated high levels of potential resilience and readiness with significant data available. Leveraging the PHRR Assessment Tool can inform investments and cross-sector partnerships that enhance companies’ readiness and resilience to a variety of public health threats. Additional research is needed to further validate this tool.

## Introduction

### Corporate public health readiness and resilience considerations

The coronavirus 2019 (COVID-19) pandemic underscored the impact that public health incidents have across individual, community, and business realms (1). For the first time, all corporations^1^ collectively experienced the significant impact that a major public health event can have to their businesses, their people, and the communities in which they operate. Executives had to manage health and safety in the workplace, deal with vacillating demand for goods and services, and manage the disruption of supply chains, all while trying to interpret fast-changing science and combat dis- and misinformation during a period of extremely high uncertainty. Additionally, many companies were forced to reevaluate their physical buildings and real estate portfolio using a “healthy building” lens for the first time (2). The pandemic called attention to how businesses are impacted by public health events and are essential actors in both community response and their employees' experiences.

The drastic economic impact that the pandemic had on businesses and communities raised questions about the potential effects of future public health events on businesses and in what ways companies can prepare and respond in the future (3). Several researchers, NGOs, governments, and companies have conducted research into public health readiness and resilience to determine best practices (4, 5). Public health readiness is an essential requirement for any company to prepare for possible public health crises or risks proactively and effectively, to minimize their impact, and to ensure rapid and comprehensive responses to protect the company's workforce, customers, suppliers, and communities. In turn, public health resilience is the capacity to adapt and respond once unforeseen public health challenges, emergencies, or disasters arise while ensuring uninterrupted operations, protecting the safety of employees and customers, and supporting the welfare of the community swiftly and successfully (6, 7). Together, readiness and resilience enable organizations to better prepare for and respond to a myriad of threats, decreasing the potential negative impact and accelerating response time. Traditional resilience and disaster response literature has focused mainly on natural disasters and epidemics (8). This manuscript, however, focuses on resilience and readiness in the context of public health, answering the question: what does it mean for companies to be resilient and ready in this context and how can an organization effectively measure the degree to which they are prepared? While the coronavirus identified in 2019 was an infectious, air-born, respiratory disease, future public health threats could stem from a variety of causes: climate, food or agricultural, other infectious agents, impacted water supply, or a combination of sources. The breadth, depth, and diversity of potential causes lends to the notion that the best crisis response is largely dependent on the nature of the threat.

Resilience is a critical success factor for both public health and businesses when navigating uncertainties, as it requires preparedness and agility when responding to events (9). Public health readiness and resilience, and the associated measures of an organization's relative maturity in these domains will depend on the nature of each threat. Some organizations will be well-positioned for specific threats and poorly prepared for others. However, most companies do not routinely self-evaluate their readiness for and likely resilience to a wide array of potential public health threats. There is a need for a standardized self-evaluation framework, comprised of dimensions that align with potential response and readiness to a broad range of public health risks (that are inclusive of and expansive beyond disaster recovery planning), which is not only relevant to but also actionable by corporate decision-makers.

To this end, we first researched, assessed, and defined dimensions of resilience for businesses through a public health lens by building on existing frameworks where possible and creating new dimensions to fill gaps where needed. We then explored dimensions of resilience from a public health risk-specific lens and reviewed existing evaluation tools and frameworks to develop a methodology and framework for organizations to evaluate themselves against. This includes critical dimensions of public health readiness and the associated criteria and metrics to accurately assess an organization's likely resilience moving forward. We then leveraged the framework to evaluate a sample of large corporations to assess how the tool functioned, the insights it could provide, and the ability to leverage publicly available vs. proprietary data to complete the assessment. In this paper, we will present the resulting assessment tool, how it was developed, and key initial findings from real-world companies.

## PART I

## Method

### Defining public health resilience from a corporate perspective and developing the PHRR assessment tool

First, we performed an extensive literature review related to the topic of resilience, how it was being defined and measured in different settings, and if any specific research was available related to public health resilience. This informed the working definition of resilience explored in stakeholder workshops and then leveraged during the rest of the tool development and testing. We then conducted an extensive search for relevant publicly available resilience tools and frameworks related to measuring levels of employee health & well-being, place-based health indicators, and preparedness. We identified and analyzed 24 existing resilience and public health readiness tools to assess the current landscape, including best practices and components that could be further built out. Each tool was assessed based on its description, purpose, category, input(s) and output(s), accessibility, and scalability. Tools were also categorized by five different elements: (1) relevance, (2) methodology, (3) ease of use, (4) output applicability, and (5) input dimensions.^2^

Our team then conducted two online workshops between October and November of 2021 to capture deeper qualitative insights from thought leaders related to resilience, tools, evaluation dimensions, and unmet market needs. Workshop attendees were selected based on their academic and professional expertise in the field of public health, policy, business, or resilience and preparedness. Both workshops had the same attendees and were manually transcribed by research assistants and later summarized. The first workshop aligned the purpose and goals of a public health resilience tool or framework and defined business resilience from a public health perspective. Participants also reviewed existing evaluation frameworks to understand existing best practices and gaps. This included exploring questions like: What does it mean to be resilient in public health crises now and in the future? How can businesses be better prepared for future health impacts on their business? What is a business' responsibility in preparing and responding to health-related crises? What does it mean for a business or company to be resilient from a public health perspective?

The second workshop focused on validating relevant public health resilience dimensions. During the session, attendees were encouraged to consider the public health rationale [i.e., why is this (dimension) important for public health?] and the business rationale [i.e., why is this (dimension) important for businesses?] for each suggested dimension to ensure relevance.

We then conducted a second literature review focused on = resilience dimensions identified in the first stage of research. These dimensions were refined and validated during the second workshop by discussing their validity and identifying multiple criteria that could be used to measure each dimension. The metrics were then tested with several business leaders to determine applicability and relevance. Once metrics were identified, we then reviewed data source availability for each one to determine what data was publicly available, what data was available for purchase, and what data would require primary research with companies to capture. Finally, after reviewing each dimension and metric used within available assessment tools and leveraging expert input on relevance to corporate public health resilience assessment, six dimensions were identified for inclusion in the draft Public Health Readiness and Resilience (PHRR) assessment tool.

## Results

### Learnings from existing evaluation tools

The 24 existing tools reviewed emphasized different aspects of public health. Examples of focus areas include organizational support(s), leadership, and company culture; strategic processes for planning public health agendas and associated impacts to the organization; specific health programs; company policies that consider employee and organizational-level public health impact as well as public health-related interactions with external partners; integration with the local public sector, and local county health metrics to understand and assess the health of the communities they work in and from where their employees reside.

However, none of the tools included all five elements (i.e., relevance, methodology, ease of use, output applicability, and input dimensions) that were identified as critical to a public health resilience-oriented framework. The majority did not incorporate a mechanism to conduct comparisons across companies or include considerations for interactions between companies and their surrounding communities. Additionally, none of the tools assessed evaluated multiple components of a company's public health resilience, such that the tool could assess the relationships between business, community, and individual employee impacts and provide a comprehensive understanding of corporate public health resilience.

Overall, the evaluated tools are valuable for measuring specific aspects of public health resilience, but none provided a complete assessment. Each contain certain elements of what businesses need to assess to understand their potential preparedness for and resilience to adverse public health events but does not provide a comprehensive or complete view. Additionally, there was a gap in the focus on companies vs. communities. Most tools assessed were developed for the purposes of community and government use rather than evaluating businesses as a comprehensive, interlocking unit. Results from the landscape assessment and thought leader workshops support the need for a novel and actionable assessment framework that leverages objective and comparable metrics relevant to current and future public health crises in a corporate setting.

### Building the public health readiness and resilience assessment tool

Six distinct dimensions emerged as priorities for a novel PHRR assessment tool. These include (1) Community Connectivity, (2) Leadership & Trust, (3) Employee Health & Well-being, (4) Operations, (5) Physical Environment, and (6) Internal Analytics & Assessment (see Table 2). In building the assessment tool, each dimension was further contextualized through specific subservient-related metrics that roll up into a collective view, and together create the composite assessment of organizational readiness and estimated resilience. Dimension definitions and assessment criteria are detailed below:

1) **Community Connectivity:** The degree to which an organization has active engagement and commitment to the local community's health and societal resilience.
   • Example of **Community Connectivity**: Continued engagement with community leaders allows for the identification of local priorities, relationship building, and bidirectional information flows that can be leveraged during times of crisis.
2) **Leadership & Trust:** The degree to which leadership is set up to prepare for, monitor, and respond to Public Health future scenarios, and the level of trust and engagement employees have with the company.
   • Example of **Leadership & Trust:** A company has well-established communication cadence with employees and has developed a high level of trust related to routine issues as well as communicating challenges.
3) **Employee Health & Well-being**: The degree to which employers ensure appropriate access and coverage to health benefits (e.g., medical benefits, paid leave policies, wellness programs, etc.) and prioritize worker health & well-being.
   • Example of **Employee Health & Well-being:** Company has embedded health and wellness into core HR activities and has a team actively deploying health and wellness initiatives among employees, actively surveying employees about their health and wellness needs, and evaluating the impact of deployed programs.
4) **Operations:** The degree to which an organization ensures that operational procedures (e.g., supply chain, IT) are repeatable and scalable.
   • Example of **Operations:** The company has a health risk monitoring system that provides ongoing insights to the operations team that conducts planning for a variety of health risk-based scenarios. They evaluate the impact on vital business functions, supply chains, services, and people. They establish an operational response to a variety of scenarios and potential challenges.
5) **Physical Environment:** The degree to which an organization focuses on the environmental and climate impact (i.e., physical environment resilience to climate threats such as sea-level rise, floods, heat, wildfire) of business activities as it pertains to health impacts, as well as the indoor environment (i.e., healthy buildings) and carbon impacts (e.g., fossil fuel consumption vs. electric usage).
   • Example of **Physical Environment:** The company reviews its physical workspaces and physical plants from a health and safety standpoint, considering both the impact of environmental or climate risks and a variety of health-related risks like air or food-borne pathogens. Specifically, they might invest in resilience from weather and climate-related impacts, as well as air quality, water, sanitation, and waste management.
6) **Internal Analytics & Assessment:** The degree to which an organization has the capability to use and actively leverage, internal and external assessments and data to drive decision-making and quantify their PHR impact.
   • Example of **Internal Analytics & Assessments:** The company operationalizes corporate data to generate forecasting, foresight, predictions, or detection of adverse events, potential system vulnerabilities, uncertainties, deteriorations, etc., for constant awareness of and preparation for current public health events.

**Table 1:** Evaluation dimensions for landscape assessment.

Evaluation Dimensions	Definition
Relevance	The tool has components that holistically consider public health resilience through a business lens
Methodology	The tool was created using a clear, repeatable, and objective methodology
Ease of use	The tool can be easily accessed and/or used
Output applicability	The tool's outputs are easily interpretable, and can drive public health resilience in business
Input dimensions	The tool's input dimensions were relevant to public health resilience in business

**Table 2:** Proposed PHRR tool dimensions.

Dimension	Key Questions	Metrics	Scoring
Community Connectivity	• How are you connected or engaged to the communities you work or serve? • Does your organization's practices and policies consider or integrate with the surrounding community?	1. Policy for community involvement	LMH
		2. Community lending and investments	LMH
		3. Ability to share information within local community	
		4. Public health monitoring and evaluation in local community	LMH
		5. Number of current community partnerships	LMH
Leadership & Trust	• Are your leaders set up to understand when public health risks are emerging and how to best respond in various scenarios? • Do you have a single leader accountable to drive response during public health crisis? • Do what degree do employees trust and engage with your company?	1. Employee satisfaction rate	LMH
		2. Average employee length of service	LMH
		3. Turnover rate	LMH
		4. Training related to health or DEI	Y/N
		5. Availability of technology trainings	LMH
		6. Funding approval pathways during emergency	Y/N
		7. Routine reviews by senior leadership of various public health risk metrics	Y/N
		8. Specified leader in charge of public health risk assessment and response	Y/N
		9. Succession plan for management	Y/N
Employee Health & Well-being	• Are you investing in your employees’ health and well-being? What programs and policies do you have in place? • Do your employees feel supported? (access to care, social networks)	1. Transparency about and awareness of available resources	LMH
		2. Occupational diseases rate	LMH
		3. Employee fatalities	LMH
		4. Flexible working hours	Y/N
		5. Day care services	Y/N
		6. Policy for employee health & safety	Y/N
		7. Policy for supply chain health & safety	Y/N
		8. Employees health & safety team	Y/N
		9. Health and safety training	Y/N
		10. Employee health & safety training hours	LMH
		11. Supply chain health & safety training	Y/N
		12. Supply chain health & safety improvements	Y/N
		13. Employees health & safety management systems	Y/N
		14. HSMS certified percentage	LMH
		15. Established new wellness programs or partnership with digital wellness platforms	LMH
		16. Participation in health and wellness programs	LMH
Operations	• Are you adapting to new tech and workplace cultural preferences? • How are you investing in crisis preparedness? • Are you preparing for better response and building agility to threats?	1. Crisis management systems	Y/N
		2. Timeliness to adapt to new public health context	LMH
		3. Response plan for adverse public health events	Y/N
		4. Cybersecurity sophistication	LMH
		5. Risk analytics automation	LMH
		6. Review cadence of emergency response plans	LMH
		7. Policy for customer health & safety	Y/N
		8. Food security assessment	Y/N
Physical Environment	• Are you actively monitoring air and water quality in all your buildings? • Is your physical infrastructure up to date and prepared for anticipated threats?	1. Environment risk assessment	LMH
		2. Monitoring of air quality	Y/N
		3. Monitoring of community or external risks (i.e., crime)	Y/N
		4. Evaluation of worksite safety	LMH
		5. Clean and safe water supply	Y/N
Internal Analytics & Assessments	• Do you track and monitor your health metrics? • What are some “improved resilience” outcomes?	1. Recent employee health & safety controversies	LMH
		2. Recent customer health & safety controversies	LMH
		3. Recent public health controversies	LMH
		4. Broad adoption of novel technologies	LMH
		5. COVID-19 dedicated section or report	Y/N

Y/N, Yes or No binary scoring; LMH, low, medium, and high scalar scoring.

The PHRR assessment tool is comprised of 48 metrics that map to these six dimensions or priority areas. The metrics are specific measures that fall within one of the six dimensions.

## PART II

## Method

### Leveraging the PHRR tool to assess real-world companies

Twenty-two companies were selected based on size (all companies had at least 4,500 employees, and the majority had 10,000+ employees) and representation across eight sectors to evaluate the applicability of the PHRR assessment tool in the real world. Companies assessed were present in multiple US states and/or other countries. The eight sectors and associated number of companies within each sector were as follows: Health sciences and wellness (*n* = 6), Consumer discretionary (*n* = 5), Financial services (*n* = 4), Energy (*n* = 2), Government and private sector (*n* = 2), Information technology (*n* = 1), Auto manufacturing (*n* = 1), and Restaurant (*n* = 1). Multi-sector representation was important due to the unique ways in which companies can be impacted by different threats. For example, while all sectors were impacted by the COVID-19 pandemic, in the early days of the crisis, the travel and restaurant industries were disproportionately impacted as compared with other industries like information technology and virtual entertainment (10). The key impacted industries of a future public health event will likely change depending on the nature and manifestation of the threat.

Once selected, each company was then assessed against the PHRR tool using publicly available data from ESG reports (i.e., disclosure of environmental, social, and corporate governance data), company websites, and other external research. The degree to which information was available for each metric determined its Metric Availability Score (MAS), with the actual metric-specific result rolling up to the Dimension Resilience Score (DRS) for each of the six dimensions. The DRS is calculated as the sum of all scores for available metrics in a dimension divided by the highest possible score in each dimension. The MAS provided the context of where further investigation or primary research with the company was needed to get higher reliability for the DRS. Both the MAS and the DRS were scored from 1 to 3, with a score of 1 indicating no fulfillment of criteria, a score of 2 indicating fulfillment of some criteria but not all criteria, and a score of 3 indicating satisfactory fulfillment of criteria. Companies also received one Total Metric Availability Score (TMAS) and one Total Company Resilience Score (TCRS). The TMAS is the ratio of a company's available metrics to all possible metrics (*n* = 48). While the TCRS is calculated as the sum of all scores of available metrics divided by the total highest possible score of available metrics (see Table 3).

**Table 3:** Key process definitions.

Terminology	Definition
Dimension	An umbrella category containing grouped metrics. There are six dimensions in the PHRR assessment tool.
Metric	A measure of specific public health readiness and resilience criteria. There are 48 total metrics in the PHRR assessment tool.
Metric Availability Score (MAS)	The number of PHRR metrics that can be scored based on a company's publicly available data, divided by the total number of metrics in a given dimension.
Total Metric Availability Score (TMAS)	The sum of a company's total available metrics to be scored divided by the total number of metrics in the PHRR assessment tool (e.g., 48 total metrics).
Dimension Resilience Score (DRS)	The sum of a company's metric scores in a given dimension, divided by the highest possible score in a given dimension.
Total Dimension Resilience Score (TDRS)	The sum of a company's total dimension resilience scores divided by the highest possible resilience score for all metrics in the PHRR assessment tool (e.g., 144 total).

## Results

### Findings from real-world companies

After assessing 22 companies, variation in readiness and resilience, as well as the availability of data, was observed. Companies were mapped into four quadrants based on performance in both TCRS and TMAS scores (see Tables 3, 4). Half of the companies analyzed (*n* = 11) scored High TCRS/High TMAS, indicating high levels of potential resilience and readiness with significant data available. High TCRS/Low TMAS companies (*n* = 8) had high resilience and readiness scores; however, they had low data transparency, indicating the need for additional follow-up evaluation to validate.

**Table 4:** Highest number of assessed companies with high and low total company resilience scores and high metric availability scores.

	Quadrant	Companies (*n*)	Description
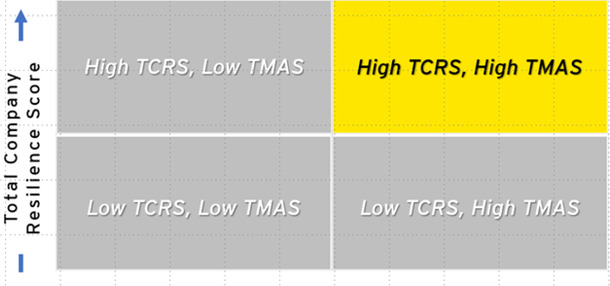	High TCRS, high TMAS *least improvement needed	11	Companies with high levels of public health resilience in all dimensions and transparency of information, meaning that information is publicly available. They are leaders in employee health and well-being, building safety, technological advances, and positive community impact. They tend to be companies in the health and wellness sector.
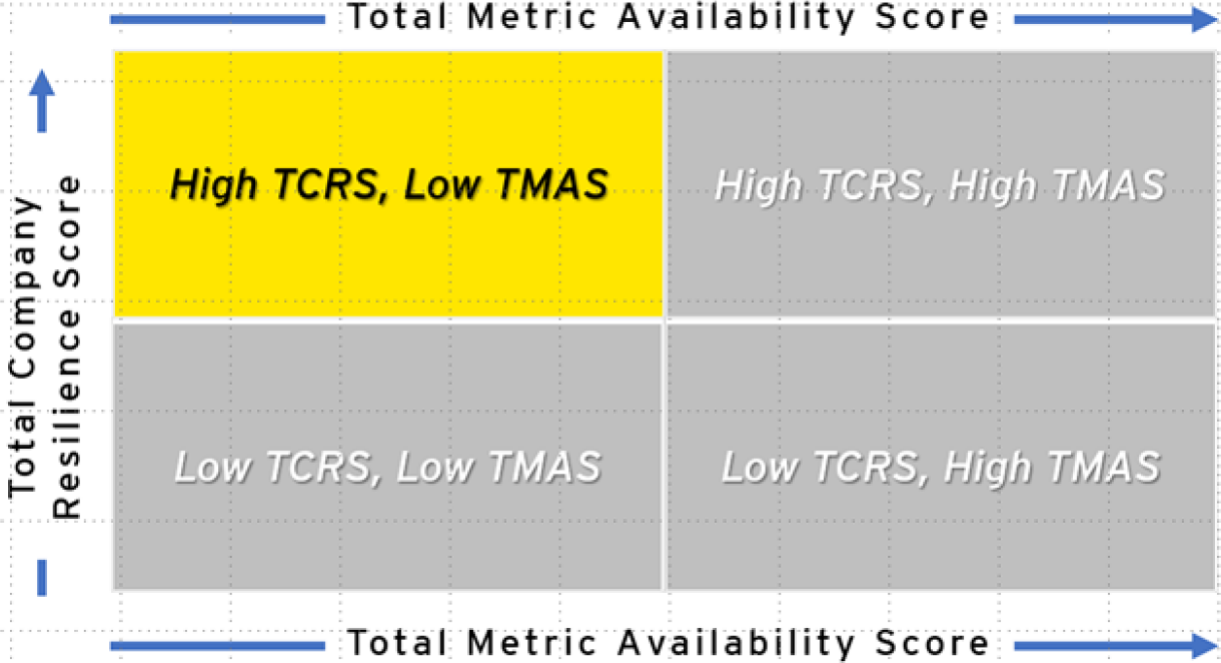	High TCRS, low TMAS	8	Companies with low transparency of information but high public health resilience. They only reveal public health information that displays achievement of key public health metrics, such as employee health and happiness and community engagement. Many of their metrics, however, did not have publicly available information online. are resilient in every dimension, especially among the employee health and well-being metrics.
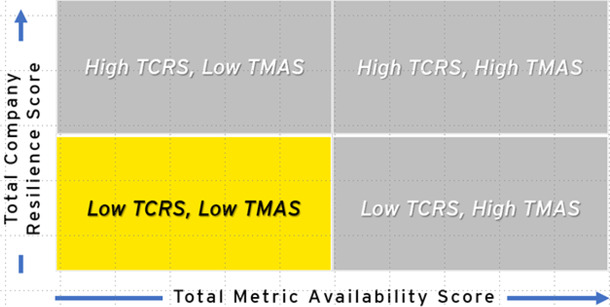	Low TCRS, low TMAS *most improvement needed	3	Companies with the lowest levels of transparency and resilience, indicate that public health information is difficult to procure, and the available information indicates less than satisfactory levels of employee health and well-being, worksite safety, technological advances, and positive community impact.
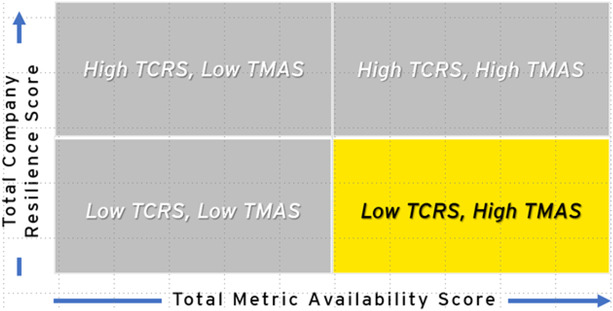	Low TCRS, high TMAS	0	Companies with low levels of resilience. As expected, we did not identify any companies that met the criteria of this quadrant. Further research is necessary to determine whether the lack of resilience is due to a lack of effort and investment or simply due to unavailable data.

Across companies, the dimensions with the least metric availability were Leadership & Trust and Operations. Eight out of 22 companies scored below 50% on Leadership and Trust, and 10 out of 22 companies scored at or below 50% on Operations, indicating poor metric availability in the Leadership and Trust and Operations dimensions. The four companies in the health sciences and wellness sector averaged 91% in their TCRS, indicating strong company resilience among health sciences and wellness companies. Across all companies, the Community Connectivity dimension had the highest scores in terms of both TMAS and TCRS displaying high company involvement with local communities, willingness to donate money, and pride in company involvement in social causes. Additionally, the companies in the government and private sector had low MAS, highlighting the need for more transparency of government public health information. The majority of companies had employee health and safety policies, employee wellness programs, and employee benefits.

Companies in the health and life sciences sectors are most likely to be public health resilient; however, because sample sizes are small in other sectors, more work is needed to fully understand the relative readiness and resilience of the broader market. Across all companies assessed there is significant room to improve public health resilience though actual corporate preparedness may be better than assessed due to a lack of publicly available information.

## Discussion

### Application and scaling of the PHRR assessment tool

The lack of comprehensive readiness and resilience assessment tools for public health threats underscores the importance of developing a novel assessment tool (i.e., PHRR assessment tool) focused on helping organizations understand where to focus efforts to become more resilient to current and future public health threats.

The goal of the PHRR assessment tool is to help businesses determine their baseline level of public health readiness and likely resilience to a variety of potential health threats based on their existing characteristics; identify vulnerabilities and opportunities to enhance; and sustain resilience through further public health focus, investment, or partnership. The PHRR assessment tool is intended to guide industry leaders to (1) Assess and improve how well their organization, as a whole, can respond to and withstand future public health crises, (2) Identify strengths and areas of improvement, with the goal of it becoming a regular assessment and monitoring tool, (3) Allow businesses to compare how they are doing with others, and (4) Share common gaps and best practices to ultimately create more uniform standards for employee well-being, community health, and enhanced productivity. This framework could also help organizations determine where to focus future investments and how to consider which ones will position them to be more resilient to current and future public health threats.

By piloting the tool with publicly available data from 22 companies within and across sectors, we began the process of validating the tool's effectiveness and value. More work is needed to continue to validate it across a broader variety of organizations and through the expanded use of both proprietary and publicly available data. The preliminary results provide actionable areas for further evaluation, and directional insights to help businesses determine where there are key gaps and actionable opportunities to enhance and sustain public health resilience through investment, focus or partnership. We envision that the tool will reside within the risk functional area, but assessment findings should be shared cross-functionally.

For the tool to be scalable, further work will be required to validate the metrics embedded in the PHRR assessment tool. Additionally, the tool could be considered from a sector-specific lens leveraging metric weighting based on the way that different factors impact companies in different sectors. This could allow each company to take a sector-specific view of its public health resilience. For example, companies in the hospitality industry would have greater weight given to employee readiness and ability to congregate freely, while the energy sector might weigh transportation, supply chain, and operational continuity higher.

Nonetheless, findings are subject to limitations, such as the fact that the professionals' personal experiences and biases may influence the information they shared with the research team, thereby limiting the findings.

## Conclusion

The COVID-19 pandemic has sharpened businesses’ focus on global threats, and resilience has become an increasingly critical concept for business to deliver economic prosperity as well as long-term community health. The ability to evaluate and measure an organization's resilience potential against a host of future public health-related risks and events is not just valuable but essential in this context. Elements of public health resilience seen in companies that have and continue to adapt to the evolving state of the coronavirus pandemic that started in 2019, such as digital communication capabilities, mature analytics, and integrated systems supporting employee health & well-being, may have been posed as forward-thinking corporate characteristics prior to the shelter-in-place policies introduced in March 2020. Today, they serve as the baseline factors for businesses that will resiliently face the public health crises of the future.

The pandemic revealed that public health and business health are inextricably linked. Yet many organizations have not incorporated public health into strategic and core business functions. Further, existing frameworks for how businesses could think about and prepare for future public health threats did not include a holistic assessment tool. Our analysis identified six key dimensions of Public Health Readiness and Resilience and associated metrics that businesses could consider for understanding and assessing public health resilience and mitigating associated business impacts from current and future threats. When companies leverage the assessment tool, findings could support investments or cross-sector collaborations to build public health and especially community resilience.

## Data Availability

The original contributions presented in the study are included in the article, further inquiries can be directed to the corresponding author.

## Footnotes

^1^A for-profit company which has been incorporated through the legal process of creating an entity or corporation (1).

^2^*Disclaimer: the tools included were not evaluated based on merit in any way and were only assessed based on whether or not they addressed the purpose of assessing public health resilience in businesses*. (Please see Table 1 for the evaluated dimensions and their definitions).
